# Health-related quality of life and associated factors among cervical cancer patients at Tikur Anbessa specialized hospital, Addis Ababa, Ethiopia

**DOI:** 10.1186/s12955-020-01319-x

**Published:** 2020-03-16

**Authors:** Liya Teklu Araya, Teferi Gedif Fenta, Beate Sander, Girma Tekle Gebremariam, Gebremedhin Beedemariam Gebretekle

**Affiliations:** 1 Social and Administrative Pharmacy Unit, School of Pharmacy, College of Health Sciences, Addis Ababa University, Zambia Street, P.O. Box: 1176, Addis Ababa, Ethiopia; 2 Institute of Health Policy, Management and Evaluation, University of Toronto, Toronto, Ontario Canada; 3 Toronto Health Economics and Technology Assessment (THETA) Collaborative, University Health Network, Toronto, Ontario Canada; 4 ICES, Toronto, Ontario Canada; 5 Public Health Ontario, Toronto, Ontario Canada; 6 Department of Pharmacology and Clinical Pharmacy, School of Pharmacy, College of Health Sciences, Addis Ababa University, Addis Ababa, Ethiopia

**Keywords:** Cervical cancer, EQ-5D, Ethiopia, Health-related quality of life, Utility

## Abstract

**Background:**

Cancer of the cervix is the most frequent cancer among women in Ethiopia. The disease burden and its treatment adversely affects patients’ health-related quality of life (HRQoL). We aimed to investigate the HRQoL and its predictors among cervical cancer patients in Ethiopia.

**Methods:**

A hospital-based cross-sectional study was conducted from January to June 2018 at the oncology unit of Tikur Anbessa Specialized Hospital, Addis Ababa, Ethiopia. A total of 404 cervical cancer patients were interviewed using validated Amharic version of the European Organization for Research and Treatment of Cancer module (EORTC QLQ-C30), cervical cancer module (EORTC QLQ-CX24), and Euro Quality of Life Group’s 5-Domain Questionnaires 5-Levels (EQ-5D) questionnaires. ANOVA test was used to determine the effect of patients’ characteristics on mean scores of the different domains of HRQoL and stepwise multivariable logistic regression was performed to identify predictors of HRQoL. Coefficients of level-specific utility values obtained from a hybrid regression model for the Ethiopian general population were used to compute utility.

**Results:**

The mean age of patients was 52.1 ± 10.4 years and 379 (93.8%) of the patients were receiving service at the outpatient clinic. About one-third (35%) of patients were diagnosed with stage IV cervical cancer. Mean global health status/QoL, mean utility and visual analog scale scores were 48.3 ± 23.77, 0.77 and 65.7 ± 20.83, respectively. Physical functioning (AOR = 4.98, 95% CI:2.16–11.49), emotional functioning (AOR = 5.25, 95% CI:2.26–12.17), pain (AOR = 5.79, 95% CI:2.30–14.57), and symptom experience (AOR = 4.58, 95% CI:1.95–10.79) were associated with patients’ HRQoL.

**Conclusions:**

Cervical cancer significantly affects patient’s HRQoL and hence, efforts to improve HRQoL should be commenced especially in terms of physical and emotional functioning, pain, and symptom experience.

## Introduction

Cervical cancer is the fourth most common cancer worldwide that affecting women with estimated incidence and mortality of 56,9847 and 31,1365, respectively, which accounts for 6.6% of all female cancers [1]. While cervical cancer survival rates are improving in developed nations, its burden in the low- and middle-income countries (LMICs) is high where nearly 90% of cervical cancer deaths are from these countries [1, 2]. The annual incidence of cervical cancer in the horn of Africa is 52,633, which leads to high mortality and morbidity [1]. The high burden of cervical cancer in LMICs is largely because the majority of cases are detected in advanced stages which in turn was ascribed to the low screening rate as a result of lack of access to health care facilities [2].

Cervical cancer is the second leading cancer among women cancer in Ethiopia. According to 2018 reports, the estimated annual number of new cervical cancer cases were 6294 while the number of cervical cancer-related deaths were 4884 annually [3]. Despite the government effort to promote screening for early diagnosis of cervical cancer, patients were diagnosed at advanced stages, leading to long periods of illness, loss of productivity and premature deaths [1]. Further, there are few oncology centers and the treatment options are limited and costly. Ethiopia has only one radiotherapy center at Tikur Anbessa Specialized Hospital and that means all patients have to travel from different regions of the country to this hospital to get the radiotherapy service [4, 5]. Therefore, cervical cancer is becoming an important health issue that affects the patient’s HRQoL [6].

HRQoL is the subjective perception of the impact of disease and treatment on the health status of the patients that encompasses domains related to physical, functional, psychosocial or emotional functioning, that used to examine the impact of disease and its treatment on HRQoL [7, 8]. It is a multidimensional and complex concept that reflects patients’ experiences with the disease and treatment [9]. Both disease and its treatment burden are known to have an impact on the quality of life. So, the assessment of HRQoL in cervical cancer patients is important in order to design interventions for improving patients’ outcomes.

Several generic and disease-specific tools have been developed for assessing HRQoL and its assessment has been made a routine practice in developed nations [10, 11]. The European Organization for Research and Treatment of Cancer module (EORTC QLQ-C30), and cervical cancer module (EORTC QLQ-CX24) are among the disease-specific tools that are most often used to evaluate HRQoL in cancer survivors [12, 13]. Generic tools are also utilized in the economic evaluations of health interventions as they provide a multidimensional description of health, which generates quality-adjusted life-years [14]. The Euro QoL 5-Dimension questionnaire (EQ-5D) is also among the widely used generic instrument to evaluate HRQoL [15]. We aimed to evaluate the HRQoL and its associated factors in patients with cervical cancer in Ethiopia.

## Methods

### Study design and participants

A hospital-based cross-sectional study was conducted among cervical cancer patients who visited Tikur Anbessa Specialized Hospital from January to June 2018. A total of 404 patients with cervical cancer were approached for the interview. Patients were considered eligible if they had a pathologically diagnosed cervical cancer, were 18 years or older, were able to understand and answer the questions, and were able to speak and communicate well in the local (Amharic) language. Patients were excluded from the interview if they were unwilling to participate, unable to understand questionnaires as decided by their physician as well as had a cognitive or mental problem.

### Instruments

We selected generic and disease-specific instruments, which are commonly used to measure HRQoL of patients with cancer, and permission to use the instruments was obtained from the respective institutions. We used three different validated instruments: EQ-5D-5 L, EORTC QLQ-C30 and EORTC QLQ-CX24 module (supplementary file-1). Details of each tool and sub-domains are described below.

#### EORTC-QLQ-C30

The 30-items EORTC QLQ-C30 is psychometrically robust, cross-culturally accepted and most frequently used tool to assess HRQoL. It is grouped into 15 domains, including five functional subscales (physical functioning, role functioning, emotional functioning, cognitive functioning, and social functioning); three multi-item symptom subscales (fatigue, nausea/vomiting, and pain); global health status/QoL subscale; and six single items addressing various symptoms and perceived financial impact [12].

#### EORTC-QLQ- CX24

The 24-items EORTC QLQ-CX24 classified into three multi-item scales, eleven items with symptom experience domain, three items with body image domain, and four items with sexual/vaginal functioning domain. The other domains of the questionnaire are single-item scales, including lymphedema, peripheral neuropathy, menopausal symptom, sexual worry, sexual activity, and sexual enjoyment [13].

#### EQ-5D

EQ-5D is a standardized measure of health status developed by the EuroQol Group in order to provide a simple, generic measure of health status for clinical and economic evaluations. It has five dimensions (mobility, self-care, usual activities, pain/discomfort, and anxiety/depression) with a five-point scale (“Not at all” to “Extreme”). The visual analog scale (VAS) measures the patient’s self-reported health on a 20 cm vertical scale, which ranges from 100 (best imaginable health state) to 0 (death or worst imaginable health state) [14–17].

### Data collection procedures

Patients who fulfilled the eligibility criteria were enrolled consecutively during their visit to the oncology clinic and a face-to-face interview was carried out to fill the questionnaires. Socio-demographic and clinical characteristics of the patients were extracted from the medical charts. Two trained oncology nurses collected the data with daily supervision by the lead investigator. Prior to data collection, the data collectors clearly explained the purpose of the study and what is expected from them. Patients were informed that their responses will not influence the management of their case and an informed consent script which was approved by the ethics committee was read to them by the nurses. Participants were also assured about the confidentiality and anonymity of the information obtained. We omitted personnel identifiers throughout the data collection and, collected data was stored in a lockable cabinet and data entered into SPSS was password protected, access to data was restricted to the research team only, and data were reported in aggregate.

### Statistical analysis

Descriptive statistics were used to report the patients’ characteristics. The coefficients of level-specific utility values obtained from a hybrid regression model for the Ethiopian general population were used to compute the utility value. Mean scores were calculated and ANOVA test was employed to examine the significance of mean difference between variables. Stepwise multivariable logistic regression analysis was used to explore the predictors of HRQoL. During bivariate analysis, all variables with *p* < 0.25 and clinically significant variables were included for multivariable logistic regression. For the purpose of analysis, we dichotomized the variables into: functional domains and global health status/QoL score of < 75 (above 75 indicates “no problem at all”) were considered as “affected at any degree” (i.e. poor functioning and global health status/QoL) whereas for symptom domains a score > 25 (below 25 indicates “no symptom at all”) were considered as “affected at any degree”. Microsoft Excel was used to calculate the EQ-5D index score but all other statistical analyses were performed using SPSS version 23. All hypotheses involved were two-sided tests; *p* < 0.05 was considered statistically significant.

## Results

### Sociodemographic and clinical characteristics

A total of 404 patients participated in the study and their mean age was 52.1 ± 10.4 years. More than half (52%) of the patients were married and 379 (93.8%) were managed as outpatients. During the study period, 219 (54.2%) of the patients were off cancer treatment and 157 (38.9%) were on radiotherapy. The duration of cancer diagnosis was less than one year in the majority (64.6%) of patients and 144 (35.60%) of the patients were diagnosed with the International Federation of Gynecology and Obstetrics (FIGO) stage IV (Table 1).

**Table 1:** Sociodemographic and clinical characteristics of the patients with cervical cancer at Tikur Anbessa Specialized Hospital, Addis Ababa, Ethiopia

Variables	Number of patients (%)
**Age category**	
25–54	230 (56.9)
55–64	120 (29.70)
> 65	54 (13.3)
**Marital status**	
Single	5 (1.2)
Married	210 (52)
Divorced	69 (19.1)
Widowed	11 (29.0)
Religion	
Orthodox	264 (65.3)
Protestant	78 (19.3)
Muslim	54 (13.4)
Others	2 (0.5)
**Educational status**	
Unable to read and write	281 (69.6)
Informal education	28 (6.9)
Primary education	42 (10.4)
Secondary education	37 (9.2)
Higher education	16 (4)
**Occupation status**	
Government	23 (5.7)
Private	10 (2.5)
Merchant	41 (10.1)
Retired	9 (2.2)
Farmer	144 (35.6)
Housewife	164 (40.6)
Others^a^	13 (3.2)
**Average monthly household income (Ethiopian Birr)**	
< 600	177 (43.8)
> 600	217 (53.7)
**FIGO Stage**	
Unknown	11 (2.70)
Stage I	10 (2.5)
Stage II	132 (31.90)
Stage III	108 (26.70)
Stage IV	144 (35.60)
**Duration of diagnosis**	
< 12 months	261 (64.6)
1–5 years	130 (32.2)
> 5 years	12 (3.0)
**Comorbid conditions**	
None	345 (85.4)
Hypertension	20 (5.0)
HIV/AIDS	28 (6.9)
Others^b^	10 (2.4)
**Treatment taken**	
None	133 (32)
Surgery	15 (3.7)
Chemotherapy	16 (4.0)
Radiotherapy	171 (42.3)
Surgery and Chemotherapy	10 (2.5)
Chemotherapy and Radiotherapy	38 (9.4)
Surgery, Chemotherapy and Radiotherapy	21 (5.2)

Others^a^ = Housemaids, commercial sex workers and homeless

Others^b^ = Diabetes Milletus, Peripheral Neuropathy, Anaemia

### Health-related quality of life

The global health status/QoL mean score was 48.3 ± 23.77. The EORTC QLQ-C30 multiple and single-item scales were also calculated and the mean score of the functional scales ranged from 40.38 ± 30.93 to 79.8 ± 26.12, the least being social functioning and the highest cognitive functioning. For the symptom scales, with the exception of diarrhea (7.43 ± 21.73), the other entire items indicated moderate to high symptoms, of which financial difficulty was the highest (68.89 ± 35.42). On the other hand, the EORTC QLQ-CX24 domains mean score ranged from 6.12 ± 19.70 for sexual activity to 64.56 ± 29.75 for sexual/vaginal functioning. On the symptom scales, the least mean score (12.76 ± 27.7) was on lymphedema and the highest was menopausal symptom (55.77 ± 35.64) (Table 2).

**Table 2:** Mean (SD) of EORTC QLQ-C30 and EORTC QLQ-CX24 of patients with cervical cancer at Tikur ASH, Addis Ababa, Ethiopia

	EORTC QLQ- C30	Item Numbers	Mean **+** SD
**EORTC QLQ-C30**	**Global health status/QoL**	29, 30	48.3 ± 23.77
	**Functional domains**		
	Physical functioning	1–5	53.0 ± 26.05
	Role functioning	6,7	47.15 ± 34.05
	Emotional functioning	21–24	57.13 ± 34.50
	Cognitive functioning	20,25	79.8 ± 26.12
	Social functioning	26,27	40.38 ± 30.93
	**Symptom domains**		
	Fatigue	10,12,18	57.2 ± 28.1
	Nausea and vomiting	14,15	21.7 ± 30.12
	Pain	9,19	59 ± 30.07
	Dyspnea	8	30.8 ± 32.10
	Insomnia	11	46.69 ± 39.25
	Loss of appetite	13	54.45 ± 39.28
	Constipation	16	53.96 ± 40.78
	Diarrhea	17	7.43 ± 21.73
	Financial difficulty	28	68.89 ± 35.42
**EORTC QLQ-CX24**	**Functional domains**		
	Body image	45–47	50.27 ± 38.76
	Sexual activity	49	6.12 ± 19.70
	Sexual enjoyment	54	44.7 ± 30.37
	Sexual/Vaginal functioning	50–53	64.56 ± 29.75
	**Symptom domains**		
	Symptom experience	31–37,38,41–43	42.59 ± 22.5
	Lymphedema	38	12.76 ± 27.7
	Peripheral neuropathy	40	42.39 ± 39.27
	Menopausal symptom	44	55.77 ± 35.64
	Sexual worry	48	55.77 ± 35.64

### Mean score differences of EORTC QLQ-C30 and QLQ-CX24 scales with demographic and clinical characteristics

It was observed that as the stage of cancer increased, the functional and symptom scales of EORTC QLQ-30 as well as the global health status/QoL had a significant mean difference among sociodemographic and clinical characteristics. Fatigue, pain, and loss of appetite resulted significant mean differences across FIGO stages. Patients who had been diagnosed 5 years prior, showed a significantly low mean on fatigue, pain, loss of appetite and financial difficulty scales. Mean differences between body image with demographic and clinical characteristics were significant. Symptom experience and peripheral neuropathy also showed significant mean difference among the different stages of cervical cancer. Details are presented in supplementary file-2.

**Fig. 1:**
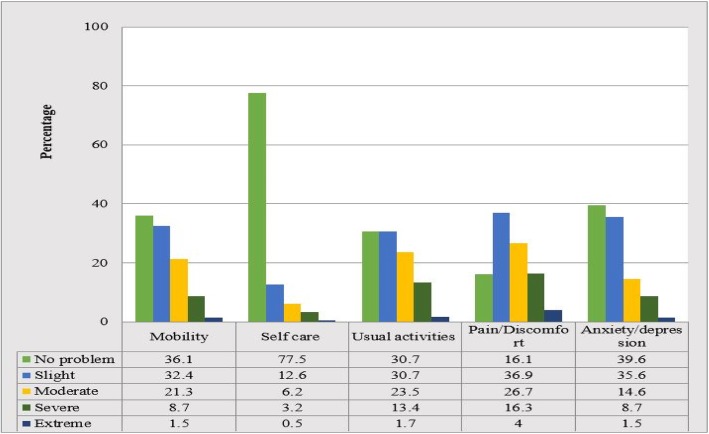
Descriptive results of EQ-5D dimensions among cervical cancer patients

The EQ-5D utility index and VAS mean score of cervical cancer patients were estimated to be 0.77 and 65.7 + 20.83, respectively. The health states reported by patients indicated that most of the cervical cancer patients in Ethiopia had none to moderate problems listed under the EQ-5D descriptive system dimension. About one-third (36.1%) of patients were able to walk and move without a problem; while the rest reported some to extreme problem with mobility. On the self-care dimension, the majority (77.5%) had no problem taking care of themselves and majority (69.3%) of the patients reported problem with usual activities dimension. Majority of the patients (83.90%) reported problem with pain/discomfort and approximately two-thirds (60.4%) of the patients reported slight to extreme problem of anxiety/depression (Fig. 1).

### Predictors of health-related quality of life

The multivariable model of HRQoL outcome revealed that four variables had significant association with the global health status/QoL. The global health status/QoL was significantly associated with physical functioning (AOR = 4.98, 95%CI = 2.16–11.49), emotional functioning (AOR = 5.25, 95%CI = 2.2612.17), pain (AOR = 5.79, 95%CI = 2.30–14.57) and symptom experience (AOR = 4.58, 95%CI = 1.95–10.79) (Table 3).

**Table 3:** Logistic regression analysis of global health status/QoL of cervical cancer patients with socio-demographic, clinical characteristics, EORTC-QLQ-CX24 and EORTC-QLQ-C 30 subscale variables

Variables	Global health status/QoL, n (%)		Odds Ratio (95% CI)	
	Affected	Unaffected	COR	AOR
Stage of cancer				
Stage I	7 (2.1)	3 (4.8)	1.33 (0.21–8.28)	
Stage II	100 (29.5)	29 (46.0)	1.97 (0.53–7.20)	
Stage III	91 (26.8)	17 (27.0)	3.05 (0.80–11.60)	
Stage IV	134 (39.5)	10 (15.9)	7.65 (1.91–30.63)	
Unknown	7 (2.1)	4 (6.3)	1.00	
Treatment				
None	119 (35)	14 (21.9)	3.09 (0.867–11.02)	
Chemotherapy	15 (4.4)	1 (1.6)	5.45 (0.53–55.80)	
Radiotherapy	143 (42.1)	28 (43.8)	1.85 (0.55–6.25)	
Surgery and Chemotherapy	5 (1.5)	5 (7.8)	0.36 (0.67–1.19)	
Chemotherapy and Radiotherapy	31 (9.1)	7 (10.9)	1.61 (3.94–6.58)	
Surgery, Chemotherapy and Radiotherapy	16 (4.7)	5 (7.8)	1.16 (0.25–5.33)	
Surgery	11 (3.2)	4 (6.3)	1.00	
Time since diagnosis				
Less than 12 Month	227 (67)	1 (1.0)	5.45 (0.53–55.80)	
1–5 years	108 (31.9)	22 (34.4)	9.81 (2.71–35.48)	
Above 5 years	4 (1.2)	8 (12.5)	1	
Physical functioning	297 (87.4)	25 (39.1)	10.77 (5.94–19.54)	4.98 (2.16–11.49)
	43 (12.6)	39 (60.9)	1.00	1.00
Role functioning	290 (85.3)	21 (32.8)	11.8 (6.50–21.68)	
	50 (14.7)	43 (67.2)	1.00	
Emotional functioning	227 (66.8)	15 (23.4)	6.52 (3.52–12.20)	5.25 (2.26–12.17)
	113 (33.2)	49 (76.6)	1.00	1.00
Cognitive functioning	134 (39.4)	10 (15.6)	3.51 (1.72–7.13)	
	206 (60.6)	54 (84.4)	1.00	
Social functioning	316 (92.9)	30 (46.9)	14.92 (7.84–28.38)	
	24 (7.1)	34 (53.1)	1.00	
Fatigue	313 (92.1)	27 (42.2)	15.88 (8.43–29.91)	
	27 (7.9)	37 (57.8)	1.00	
Nausea and vomiting	132 (38.8)	6 (9.4)	6.13 (2.57–14.61)	
	208 (61.2)	58 (90.6)	1.00	
Pain	318 (93.5)	25 (39.1)	22.54 (11.62–43.73)	5.79 (2.30–14.57)
	22 (6.5)	39 (60.9)	1.00	1.00
Dyspnea	212 (62.4)	14 (21.9)	5.91 (3.14–11.12)	
	128 (37.6)	50 (78.1)	1.00	
Insomnia	250 (73.5)	25 (39.1)	4.33 (2.48–7.5)	
	90 (26.5)	39 (60.9)	1.00	
Loss of appetite	273 (80.3)	26 (40.6)	5.95 (3.38–10.48)	
	67 (19.7)	38 (59.4)	1.00	
Constipation	256 (75.3)	25 (39.1)	4.75 (2.71–8.31)	
	84 (24.7)	39 (60.9)	1.00	
Diarrhea	47 (13.8)	2 (3.1)	4.97 (1.17–21.01)	
	293 (86.2)	62 (96.9)	1	
Financial difficulty	306 (90)	41 (64.1)	5.04 (2.71–9.39)	
	34 (10.0)	23 (35.9)	1.00	
Body image	242 (71.2)	22 (34.4)	4.71 (2.67–8.30)	
	98 (28.8)	42 (65.6)	1.00	
Sexual activity	334 (98.5)	61 (95.3)	3.28 (0.76–14.10)	
	5 (1.5)	3 (4.7)	1.00	
Sexual functioning	16 (57.1)	4 (30.8)	3.00 (0.74–12.11)	
	12 (42.)	9 (69.2%)	1.00	
Symptom experience	279 (85.8)	16 (27.6)	15.92 (8.27–30.64)	4.58 (1.95–10.79)
	46 (14.2)	42 (72.4)	1.00	1.00
Peripheral neuropathy	222 (65.3)	25 (39.1)	2.93 (1.69–5.08)	
	118 (34.7)	39 (60.9)	1.00	
Menopausal symptoms	285 (83.8)	34 (53.1)	4.57 (2.58–8.08)	
	55 (16.2)	30 (46.9)	1.00	

## Discussion

This is the first study to assess the HRQoL and factors that influence the HRQoL as well as utility among Ethiopian cervical cancer survivors. Overall, pain and anxiety/depression were the most frequently reported symptoms among patients. The different domains of the HRQoL of most patients in this study were generally poor, except diarrhea. Social and sexual functioning domains score of the EORTC QLQ-CX24 were quite lower. Physical functioning, emotional functioning, pain, and symptom experience were significantly associated with patients’ overall global health status/QoL. The finding of the present study confirmed that cervical cancer hinders not only the HRQoL but also the holistic management of cervical cancer, which urges the availability of comprehensive treatment strategies to cervical cancer patients.

The general health status/QoL mean score was low, which is consistent with Iranian and Chinese findings [18, 19], but lower than Brazilian and Indian studies [20, 21]. The possible reasons could be the cultural belief, distorted perception of survivors regarding their illness, and lower self-esteem [22]. In this study, diarrhea was among the least reported symptoms among cervical cancer survivors, which is similar to the study conducted in India [23]. However, a study done in the United Kingdom showed that diarrhea was the most frequently reported symptom [24]. This might be due to the different treatment protocols used for the management of cancer among the different countries.

The EORTC QLQ-CX24 mean scores demonstrated that younger patients have poor body image and lower sexual/vaginal functioning as compared to older patients. This finding complements the result of Bae and Park (2016), that poor body image is due to low self-esteem observed among women who have not yet children [25]. Additionally, shame and regret contributed significantly to poorer body image and lower sexual/vaginal functioning in younger age groups [26]. Social and sexual functioning scores were minimal that only 10% of the patients were sexually active, which is similar to the study conducted elsewhere [25, 26].

The finding of the present study confirmed that it is difficult for patients to interact with their partners and face problems to engage in sexual activity due to the disease and treatment burdens. The finding of the present study is consistent with the finding of Froeding et al., (2014), where almost 85% of the patients lost their sexual interest as well as 50% of the survivors complain mildly to severe pain during sexual intercourse [27]. Except for diarrhea and lymphedema domains of the EORTC QLQ-CX24, the patients demonstrated low mean scores. This is complementary with another finding, where cervical cancer survivors reported pain in the abdomen, urinary leakage, dyspareunia and menopausal symptoms [28, 29].

In the present study, about 65% of patients self-reported that their health status is primarily affected by anxiety/depression, which is similar to Scotland’s report [30]. The different dimensions of the EQ-5D affected substantially as the disease advanced. The findings indicated that the overall utility score of the patients was 0.77, which implies that patients favored to live 7.7 years with full health than 10 years with the current health condition. This showed HRQoL becomes an increasingly important concern for both patients and health care providers [25].

Physical functioning, emotional functioning, and pain, as well as symptom experience, were associated with patients’ global health status/QoL. A study done in Iran found similar predictive factors with the exception of physical functioning [24]. Thus, functioning domains are often a neglected but integral part of the HRQoL of patients that requires evaluation and treatment [31]. In Ethiopia, where women play an important role in the family that affects physical functioning, that might have a large impact on self-satisfaction and HRQoL of patients. Furthermore, inadequate infrastructure and means of transportation in the daily life of a woman from the rural area of Ethiopia becomes an additional burden for them to reach health facility, which affects physical functioning.

The patient’s global health status/QoL was highly correlated with the emotional functioning of the cancer survivors, which is in line with another study [32, 33]. The possible reason is that emotional distress, anxiety, depression affects the overall health status of the patients [34]. In complement with the above finding, Herzog and Wright (2007) found that anxiety and emotional distress greatly affected the patients’ HRQoL of cervical cancer survivors [24]. The cultural beliefs and illness perception also affect the emotion of the patients as well [31]. Hence, to reduce emotional distress among patients psychological intervention should be commenced.

This study showed that pain and symptom experience were negatively associated with global health status/QoL. Previous studies also found similar findings and emphasized that the degree of pain was directly related to global health status/QoL of patients with cervical cancer patients [25, 34, 35]. Likewise, another study indicated that symptom experiences significantly affect the global health status/QoL of patients [20]. Thus, symptom management should be an important component of patient care for cervical cancer survivors.

In contrary to other studies, the current study did not find a sexual activity as predictive of global health status/QoL of patients [18, 27]. Even though sexual activity is very low, it may not have been predictive due to the values that sexual activity in comparison to the illness. However, studies confirm that the ability to sexual activity had a positive relationship with global health status/QoL [21, 27]. The recruitment of the small number of sexually active patients might affect the true correlation of the scales; as a result, further researches with larger sample size among sexually active women should be warranted to understand the association.

The study has certain limitations. We used a cross-sectional study missing control group and there is no comparison of before and after treatment effects. Further, the study was conducted in a single setting, which might be difficult to generalize for the country. Despite the limitations, the study is the first of its kind to evaluate the HRQoL, factors affecting HRQoL and the utility value of cervical cancer patients in Ethiopia. In addition, we used validated measurement tools and recruited a large sample size. The findings can be used to design effective interventions to improve cervical cancer patient clinical outcomes. It could also help with the economic evaluation of existing and new chemotherapy medications for patients with cervical cancer.

## Conclusions

The finding of the study suggests that the HRQoL patients with cervical cancer in Ethiopian was low with mean global health status/QoL score of 48.3 ± 23.77 and EQ-5D index of 0.77. Physical functioning, emotional functioning, pain, and symptom experience significantly affects the global health status/QoL. Hence, symptom management strategies in addressing the functioning and symptoms of patients should be warranted. Furthermore, in order to recognize the disease at an early stage, screening of cervical cancer is recommended.

## Supplementary information

**Additional file 1: S1.** EORTC QLQ-C30 (version 3) and EORTC QLQ-CX24.

**Additional file 2: S2.** Mean differences of EORTC QLQ-C30 functional scale with demographic and clinical characteristics of patients at Tikur Anbessa Specialized Hospital, Addis Ababa, Ethiopia.

## Data Availability

The dataset which the study is based on is publicly not available due to required data protection but is available upon reasonable request with a signature of a data privacy form. To request the data, the reader may contact the authors through email.

## Abbreviations

ECOG-PS: European Cooperative Oncology Group Performance Status
EORTC QLQ-CX24: European Organization for Research and Treatment of Cancer Quality of life Questionnaires cervical cancer-specific module
EORTC QLQ-C30: European Organization for Research and Treatment of Cancer Quality of life Questionnaires version 30
FIGO: International Federation of Gynaecology and Obstetrics
HRQoL: Health-Related Quality of Life
TASH: Tikur Anbessa Specialized Hospital
