# Supplementary material for: Health-related quality of life and associated factors among cervical cancer patients at Tikur Anbessa specialized hospital, Addis Ababa, Ethiopia
Source: Health Qual Life Outcomes. 2020 Mar 16;18:72. doi: 10.1186/s12955-020-01319-x (PMC7076924; doi:10.1186/s12955-020-01319-x)
Supplement: Supplementary file 1 — Additional file 1: S1. EORTC QLQ-C30 (version 3) and EORTC QLQ-CX24. [file 12955_2020_1319_MOESM1_ESM.docx]

**Section 1: EORTC QLQ-C30 (version 3) and EORTC QLQ-CX24**

We are interested in some things about you and your health. Please answer all of the questions yourself by circling the number that best applies to you. There are no "right" or "wrong" answers. The information that you provide will remain strictly confidential.

| Please fill in your initials: __________  Your birthdate (Day, Month, Year): ____________  Today's date (Day, Month, Year): ____________  **EORTC QLQ-C30** | | | | |
| --- | --- | --- | --- | --- |
|  | **Not at All** | **A Little** | **Quite a Bit** | **Very Much** |
| 1. Do you have any trouble doing strenuous activities, |  |  |  |  |
| like carrying a heavy shopping bag or a suitcase? | 1 | 2 | 3 | 4 |
| 2. Do you have any trouble taking a long walk? | 1 | 2 | 3 | 4 |
| 3. Do you have any trouble taking a short walk outside of the house? | 1 | 2 | 3 | 4 |
| 4. Do you need to stay in bed or a chair during the day? | 1 | 2 | 3 | 4 |
| 5. Do you need help with eating, dressing, washing |  |  |  |  |
| yourself or using the toilet? | 1 | 2 | 3 | 4 |
| 6. Were you limited in doing either your work or other daily activities? | 1 | 2 | 3 | 4 |
| 7. Were you limited in pursuing your hobbies or other |  |  |  |  |
| leisure time activities? | 1 | 2 | 3 | 4 |
| 8. Were you short of breath? | 1 | 2 | 3 | 4 |
| 9. Have you had pain? | 1 | 2 | 3 | 4 |
| 10. Did you need to rest? | 1 | 2 | 3 | 4 |
| 11. Have you had trouble sleeping? | 1 | 2 | 3 | 4 |
| 12. Have you felt weak? | 1 | 2 | 3 | 4 |
| 13. Have you lacked appetite? | 1 | 2 | 3 | 4 |
| 14. Have you felt nauseated? | 1 | 2 | 3 | 4 |
| 15. Have you vomited? | 1 | 2 | 3 | 4 |
| 16. Have you been constipated? | 1 | 2 | 3 | 4 |

| **During the past week:**  17. Have you had diarrhea? | **Not at All**  1 | **A Little**  2 | **Quite a Bit**  3 | **Very Much**  4 |
| --- | --- | --- | --- | --- |
| 18. Were you tired? | 1 | 2 | 3 | 4 |
| 19. Did pain interfere with your daily activities? | 1 | 2 | 3 | 4 |
| 20. Have you had difficulty in concentrating on things, like reading a newspaper or watching television? | 1 | 2 | 3 | 4 |
| 21. Did you feel tense? | 1 | 2 | 3 | 4 |
| 22. Did you worry? | 1 | 2 | 3 | 4 |
| 23. Did you feel irritable? | 1 | 2 | 3 | 4 |
| 24. Did you feel depressed? | 1 | 2 | 3 | 4 |
| 25. Have you had difficulty remembering things? | 1 | 2 | 3 | 4 |
| 26. Has your physical condition or medical treatment interfered with your family life? | 1 | 2 | 3 | 4 |
| 27. Has your physical condition or medical treatment interfered with your social activities? | 1 | 2 | 3 | 4 |
| 28. Has your physical condition or medical treatment caused you financial difficulties? | 1 | 2 | 3 | 4 |

**For the following questions please circle the number between 1 and 7 that best applies to you**

1. How would you rate your overall health during the past week?

1 2 3 4 5 6 7

Very poor Excellent

1. How would you rate your overall quality of life during the past week?

1 2 3 4 5 6 7

Very poor Excellent

**EORTC QLQ – CX24:** Patients sometimes report that they have the following symptoms or problems. Please indicate the extent to which you have experienced these symptoms or problems, please answer by circling the number that best applies to you.

| **During the past week:** | **Not at all** | **A little** | **Quite a bit** | **Very much** |
| --- | --- | --- | --- | --- |
| 31. Have you had cramps in your abdomen? | 1 | 2 | 3 | 4 |
| 32. Have you had difficulty in controlling your bowels? | 1 | 2 | 3 | 4 |
| 33. Have you had blood in your stools (motions)? | 1 | 2 | 3 | 4 |
| 34. Did you pass water/urine frequently? | 1 | 2 | 3 | 4 |
| 35. Have you had pain or a burning feeling when passing water/urinating? | 1 | 2 | 3 | 4 |
| 36. Have you had leaking of urine? | 1 | 2 | 3 | 4 |
| 37. Have you had difficulty emptying your bladder? | 1 | 2 | 3 | 4 |
| 38. Have you had swelling in one or both legs? | 1 | 2 | 3 | 4 |
| 39. Have you had pain in your lower back? | 1 | 2 | 3 | 4 |
| 40. Have you had tingling or numbness in your hands  or feet? | 1 | 2 | 3 | 4 |
| 41. Have you had irritation or soreness in your vagina  or vulva? | 1 | 2 | 3 | 4 |
| 42. Have you had discharge from your vagina? | 1 | 2 | 3 | 4 |
| 43. Have you had abnormal bleeding from your vagina? | 1 | 2 | 3 | 4 |
| 44. Have you had hot flushes and/or sweats? | 1 | 2 | 3 | 4 |
| 45. Have you felt physically less attractive as a result of your disease or treatment? | 1 | 2 | 3 | 4 |
| 46. Have you felt less feminine as a result of your disease or treatment? | 1 | 2 | 3 | 4 |
| 47. Have you felt dissatisfied with your body? | 1 | 2 | 3 | 4 |

| **During the past 4 weeks:** | **Not at all** | **A little** | **Quite a bit** | **Very much** |
| --- | --- | --- | --- | --- |
| 48. Have you worried that sex would be painful? | 1 | 2 | 3 | 4 |
| 49. Have you been sexually active? | 1 | 2 | 3 | 4 |
| **Answer these questions only if you have been** | **Not** | **A** | **Quite** | **Very** |
| **sexually active during the past 4 weeks:** | **at all** | **little** | **a bit** | **much** |
| 50. Has your vagina felt dry during sexual activity? | 1 | 2 | 3 | 4 |
| 51. Has your vagina felt short? | 1 | 2 | 3 | 4 |
| 52. Has your vagina felt tight? | 1 | 2 | 3 | 4 |
| 53. Have you had pain during sexual intercourse or other sexual activity? | 1 | 2 | 3 | 4 |
| 54. Was sexual activity enjoyable for you? | 1 | 2 | 3 | 4 |

**Section 2: EQ-5D-5L**

Under each heading, please tick the ONE box that best describes your health TODAY.

# MOBILITY

I have no problems in walking about 

I have slight problems in walking about 

I have moderate problems in walking about 

I have severe problems in walking about 

I am unable to walk about 

# SELF-CARE

I have no problems washing or dressing myself 

I have slight problems washing or dressing myself 

I have moderate problems washing or dressing myself 

I have severe problems washing or dressing myself 

I am unable to wash or dress myself 

**USUAL ACTIVITIES** *(e.g. work, study, housework, family or leisure activities)*

Sample

I have no problems doing my usual activities 

I have slight problems doing my usual activities 

I have moderate problems doing my usual activities 

I have severe problems doing my usual activities 

I am unable to do my usual activities 

# PAIN / DISCOMFORT

I have no pain or discomfort 

I have slight pain or discomfort 

I have moderate pain or discomfort 

I have severe pain or discomfort 

I have extreme pain or discomfort 

# ANXIETY / DEPRESSION

I am not anxious or depressed 

I am slightly anxious or depressed 

I am moderately anxious or depressed 

I am severely anxious or depressed 

I am extremely anxious or depressed

We would like to know how good or bad your health is TODAY.

- This scale is numbered from 0 to 100.
- 100 means the best health you can imagine. 0 means the worst health you can imagine.
- Mark an X on the scale to indicate how your health is TODAY.
- Now, please write the number you marked on the scale in the box below.

The best health you can imagine

100

95

90

85

80

75

70

65

60

55

**YOUR HEALTH TODAY =**

50

45

40

35

30

25

20

15

10

5

0
