# Supplementary material for: Health-related quality of life and associated factors among cervical cancer patients at Tikur Anbessa specialized hospital, Addis Ababa, Ethiopia
Source: Health Qual Life Outcomes. 2020 Mar 16;18:72. doi: 10.1186/s12955-020-01319-x (PMC7076924; doi:10.1186/s12955-020-01319-x)
Supplement: Supplementary file 2 — Additional file 2: S2. Mean differences of EORTC QLQ-C30 functional scale with demographic and clinical characteristics of patients at Tikur Anbessa Specialized Hospital, Addis Ababa, Ethiopia. [file 12955_2020_1319_MOESM2_ESM.docx]

**Table 1: Mean differences of EORTC QLQ-C30 functional scale with socio-demographic and clinical characteristics of patients at TASH, Addis Ababa, Ethiopia.**

|  |  | GQoL | PF | RF | EF | CF | SF |  |  |
| --- | --- | --- | --- | --- | --- | --- | --- | --- | --- |
| Age(years) | 25-54 | 47.97±23.17 | 52.28±26.42 | 45.07±34.37 | 56.41±33.97 | 79.85±26.1 | 38.11±30.81 |  |  |
|  | 55-64 | 48.68±23.88 | 52.33±25.74 | 48.33±34.19 | 58.68±35.11 | 80.41±25.71 | 42.77±30.38 |  |  |
|  | >65 | 49.53±26.37 | 57.53±28.52 | 53.39±35.37 | 56.79±36.30 | 78.7±27.55 | 44.75±32.34 |  |  |
|  | *p*-value | 0.89 | 0.40 | 0.25 | 0.84 | 0.82 | 0.22 |  |  |
| Marital status | Single | 70.0±18.25 | 65.33±25.99 | 80±21.73 | 66.66±33.33 | 83.33±23.57 | 53.33±44.72 |  |  |
|  | Married | 49.04±23.18 | 53.68±25.39 | 48.57±34.4 | 55.99±34.59 | 80.79±25.44 | 38.01±30.02 |  |  |
|  | Divorced | 46.25±21.46 | 53.43±27.26 | 42.75±32.27 | 61.11±31.30 | 79.46±26.68 | 38.4±25.77 |  |  |
|  | Widowed | 47.72±26.22 | 50.88±28.34 | 45.58±36.11 | 55.84±36.62 | 78.2±27.37 | 45.15±33.97 |  |  |
|  | p-value | 0.17 | 0.57 | 0.10 | 0.64 | 0.84 | 0.15 |  |  |
| Level of education | Can’t read and write | 44.6±23.33 | 50.84±26.43 | 43.95±33.67 | 53.32±35.52 | 77.69±27.22 | 35.88±28.73 |  |  |
|  | Informal education | 53.57±24.88 | 55.95±25.68 | 45.83±33.83 | 63.39±31.86 | 88.09±18.06 | 41.66±35.28 |  |  |
|  | Primary education | 54.16±22.56 | 58.25±25.96 | 53.17±33.58 | 61.7±31.88 | 81.34±24.74 | 53.57±33.64 |  |  |
|  | Secondary education | 58.78±18.73 | 57.29±27.05 | 54.5±36.14 | 73.64±25.34 | 87.83±21.74 | 51.35±31.02 |  |  |
|  | Higher education | 66.66±25.27 | 62.08±27.18 | 72.91±36.95 | 63.02±35.61 | 81.25±27.13 | 57.29±34.4 |  |  |
|  | P value | *0.00** | *0.15* | *0.01** | *0.01** | *0.79* | 0.0* |  |  |
| AMHI in Ethiopian Birr | ≤600 | 46.79±24.95 | 52.35±26.42 | 44.91±34.53 | 54.80±35.54 | 77.96±26.78 | 39.07±31.48 |  |  |
|  | >600 | 50.15±22.72 | 53.85±26.33 | 49.23±34.31 | 59.56±33.46 | 81.72±25.12 | 41.02±30.68 |  |  |
|  | *p*-value | 0.16 | 0.57 | 0.22 | 0.17 | 0.15 | 0.36 |  |  |
| Time since diagnosis (months)  Stage of cancer | <12 | 47.54±23.5 | 54.25±25.4 | 46.61±34.1 | 57.18±34.6 | 80.90±25.6 | 38.12±30.1 |  |  |
|  | 13-60 | 47.24±22.4 | 48.71±27.9 | 45.76±34.4 | 55.25±34.2 | 76.66±27.3 | 41.79±30.09 |  |  |
|  | >61 | 77.77±25.2 | 73.33±22.3 | 69.44±35.4 | 74.30±35.07 | 90.27±20.6 | 69.44±38.8 |  |  |
|  | *p*-value | 0.00* | 0.04* | 0.07 | 0.19 | 0.12 | 0.02* | 0.02* |  |
|  | Unknown | 56.81±35.7 | 59.39±34.9 | 48.48±43.1 | 65.15±34.3 | 86.36±20.8 | 45.45±42.8 |  |  |
|  | Stage 1 | 65±19.9 | 67.33±25.0 | 76.66±27.4 | 70±28.38 | 83.33±22.2 | 75±28.5 |  |  |
|  | Stage 2 | 52.01±24.6 | 53.48±28.6 | 49.74±37.8 | 56.07±35.5 | 80.74±25.7 | 44.96±33.9 |  |  |
|  | Stage 3 | 50.46±22.4 | 56.79±24.4 | 50.61±32.4 | 61.88±31.7 | 84.56±22.0 | 42.74±29.2 |  |  |
|  | Stage 4 | 41.60±21.4 | 48.24±24.9 | 39.58±30.8 | 52.77±35.8 | 75.11±29.1 | 31.59±25.2 |  |  |
|  | *p*-value | *0.00** | *0.03** | *0.00** | *0.17* | *0.05** | *0.00** |  |  |
| Current treatment | Surgery | 59.44±25.5 | 71.55±24.3 | 67.77±38.5 | 60±30.0 | 90±16.4 | 60±36.6 |  |  |
|  | Chemotherapy | 42.70±23.1 | 47.08±22.3 | 41.66±31.6 | 59.89±34.1 | 84.37±17.7 | 30.20±23.7 |  |  |
|  | Radiotherapy | 48.53±23.0 | 52.86±25.4 | 45.61±33.7 | 57.55±34.8 | 76.80±28.3 | 38.49±29.6 |  |  |
|  | Surgery & chemotherapy | 64.16±32.2 | 61.33±34.3 | 51.66±43,3 | 64.16±43.0 | 90±31.62 | 66.66±40.8 |  |  |
|  | Chemotherapy & radiation | 50.87±21.6 | 51.75±30.5 | 53.07±37.5 | 57.45±33.9 | 80.70±20.6 | 53.07±32.3 |  |  |
|  | Surgery, radiation & chemotherapy | 58.75±26.8 | 59.66±22.6 | 59.16±32.6 | 75±27.1 | 95±15.3 | 48.33±30.0 |  |  |
|  | None | 44.17±22.5 | 50.42±26.6 | 43.48±33.3 | 52.81±34.8 | 78.94±26.3 | 35.08±29.1 |  |  |
|  | *p*-value | *0.01** | *0.07* | *0.08* | *0.23* | *0.04** | *0.00** |  |  |
| Comorbid conditions | Yes | 44.91±23.6 | 50.28±26.9 | 39.23± 33.1 | 51.27± 35.7 | 77.40±26.2 | 40.67±29.0 |  |  |
|  | No | 48.98±23.7 | 53.46 ± 26.4 | 48.50 ± 34.5 | 58.14±34.3 | 80.28±26.1 | 40.3± 31.28 |  |  |
|  | *p*-value | 0.22 | 0.39 | 0.06 | 0.16 | 0.43 | 0.94 | 0.94 | |

*PF=Physical Functioning, RF=Role functioning, EF=Emotional functioning, CF =Cognitive functioning, SF=Social functioning,* *AMHI=Average monthly house income* **The mean difference is significant< 0.05*

**Table 2: Mean differences of EORTC QLQ-C30 symptom scale with socio-demographic and clinical characteristics of patients with cervical cancer at TASH, Addis Ababa, Ethiopia.**

| Variables | | Fatigue | Nausea/Vomiting | Pain | Dyspnea | Insomnia | Appetite loss | Constipation | Diarrhea | Financial Difficulties |
| --- | --- | --- | --- | --- | --- | --- | --- | --- | --- | --- |
| Age(years) | 25-54 | 57.29±28.4 | 25.36±32.09 | 61.44±31.2 | 32.31±32.1 | 47.39±38.9 | 55.94±39.1 | 54.78±40.7 | 8.26±22.3 | 72.02±35.0 |
|  | 55-64 | 58.33±27.4 | 18.47±30.3 | 59.02±29.3 | 31.38±32.1 | 46.94±39.7 | 51.66±38.8 | 52.22±42.2 | 5.27±19.3 | 65±35.8 |
|  | >65 | 54.73±28.7 | 13.58±26.3 | 53.70±30.4 | 23.45±31.4 | 43.20±40.2 | 54.32±41.1 | 54.32±37.9 | 8.02±24.1 | 64.19±35.3 |
|  | *p*-value | 0.74 | 0.02* | 0.24 | 0.18 | 0.78 | 0.63 | 0.86 | 0.46 | 0.12 |
| Marital status | Single | 72±35.03 | 6.66±14.90 | 46.66±18.25 | 6.66±14.9 | 0.0000 | 0.0000 | 26.66±36.51 | 0.0000 | 66.66±40.28 |
|  | Married | 65±35.8 | 20.95±30.6 | 59.36±30.9 | 29.52±31.2 | 49.20±39.4 | 52.53±39.3 | 57.61±40.0 | 8.41±23.2 | 68.88±35.9 |
|  | Divorced | 64.19±35.3 | 23.43±30.2 | 60.62±29.1 | 34.29±31.8 | 45.89±38.8 | 58.45±36.3 | 49.27±41.4 | 5.79±20.5 | 72.94±33.9 |
|  | Widowed | 59.44±30.1 | 22.64±33.0 | 59.97±31.7 | 31.33±33.9 | 45.01±39.2 | 57.26±40.0 | 51.28±41.4 | 6.83±20.3 | 66.66±35.5 |
|  | *p*-value | 0.04* | 0.66 | 0.80 | 0.26 | 0.04* | 0.09 | 0.15 | 0.69 | 0.71 |
| Level of education | Can’t read and write | 60.06±26.7 | 23.54±32.0 | 62.51±29.1 | 34.40±33.4 | 48.75±39.2 | 59.78±38.4 | 55.51±40.9 | 6.88±21.4 | 71.17±34.5 |
|  | Informal education | 47.61±27.8 | 17.85±27.5 | 56.54±31.8 | 26.19±30.5 | 41.66±36.9 | 51.19±35.6 | 64.28±37.3 | 8.33±23.3 | 70.23±39.8 |
|  | Primary education | 56.61±30.8 | 21.82±33.2 | 59.12±30.8 | 20.63±26.4 | 52.38±42.3 | 39.68±39.1 | 46.03±42.2 | 11.90±26.3 | 67.46±34.9 |
|  | Secondary education | 50.45±33.9 | 14.86±25.6 | 50.0±36.8 | 25.22±26.5 | 33.33±36 | 41.44±39.6 | 50.4±40.5 | 2.7±12.1 | 57.6±34.8 |
|  | Higher education | 42.36±24.7 | 12.5±23.17 | 39.58±28.4 | 16.66±24.3 | 35.4±37.45 | 35.4±41.2 | 37.5±36.2 | 12.5±26.8 | 56.2±41.7 |
|  | *p*-value | 0.01* | 0.33 | 0.01* | 0.13 | 0.10 | 0.00* | 0.15 | 0.33 | 0.13 |
| AMHI in Ethiopian Birr | ≤600 | 59.38±28.4 | 22.78±31.7 | 62.42±30.7 | 33.89±31.6 | 49.71±39.2 | 58.00±38.4 | 54.99±41.1 | 7.53±22.0 | 69.30±35.2 |
|  | >600 | 55.19±27.7 | 21.04±30.8 | 57.14±30.5 | 28.11±31.8 | 44.39±39.2 | 50.99±39.6 | 52.22±40.7 | 7.52±21.9 | 68.35±35.7 |
|  | *p*-value | 0.14 | 0.58 | 0.09 | 0.73 | 0.18 | 0.78 | 0.50 | 0.99 | 0.79 |
| Time since diagnosis (months) | <12 | 56.87±27.2 | 22.22±31.5 | 60.08±30.1 | 30.14±32.0 | 47.89±39.3 | 55.68±39.7 | 56.44±39.7 | 6.64±20.8 | 71.00±33.8 |
|  | 13-60 | 61.19±27.9 | 21.92±30.9 | 62.56±29.7 | 33.84±32.6 | 45.89±38.9 | 55.38±37.9 | 52.05±42.3 | 9.48±24.2 | 68.20±36.3 |
|  | >60 | 25±31.1 | 11.11±22.8 | 20.83±27.6 | 13.88±22.2 | 25±35.1 | 19.44±30.0 | 22.22±35.7 | 0.00 | 36.11±41.1 |
|  | *P*-value | 0.00* | 0.48 | 0.00* | 0.10 | 0.14 | 0.01* | 0.14 | 0.24 | 0.03* |
| Stage of cancer | Unknown | 45.45±35.9 | 10.60±17.1 | 43.93±37.4 | 24.24±33.6 | 54.54±47.7 | 42.42±44.9 | 42.42±49.6 | 15.15±34.5 | 51.51±47.9 |
|  | Stage 1 | 44.44±25.6 | 5±15.8 | 35±24.1 | 13.33±17.2 | 43.33±41.7 | 33.33±41.5 | 30±42.8 | 16.66±36. | 50±42.3 |
|  | Stage 2 | 54.52±31.3 | 20.54±31.0 | 57.49±32.4 | 28.68±32.7 | 44.96±39.0 | 46.51±40.9 | 52.71±41.3 | 5.94±17.4 | 64.08±37.4 |
|  | Stage 3 | 56.27±27.3 | 21.14±30.5 | 55.24±30.0 | 28.70±30.3 | 43.82±36.6 | 54.93±37.3 | 51.54±40.6 | 7.40±23.3 | 70.67±35.2 |
|  | Stage 4 | 62.5±24.4 | 25.11±32.8 | 68.63±26.3 | 36.34±32.9 | 50.69±40.5 | 63.88±36.6 | 59.72±38.9 | 7.40±21.7 | 74.53±31.2 |
|  | p-value | 0.04* | 0.18 | 0.00* | 0.07 | 0.59 | 0.01* | 0.10 | 0.44 | 0.02* |
| Current treatment | Surgery | 40±29.9 | 6.66±18.6 | 36.66±32.8 | 26.66±38.2 | 17.77±27.7 | 37.77±45.1 | 31.11±36.6 | 0.0000 | 60±38.21 |
|  | Chemotherapy | 63.19±25.2 | 32.29±39.1 | 70.83±22.3 | 41.66±31.0 | 43.75±37.9 | 62.5±38.2 | 66.66±29.8 | 2.08±8.3 | 85.41±17.0 |
|  | Radiotherapy | 57.11±28.5 | 23.09±31.9 | 59.25±31.5 | 31.57±32.3 | 46.39±39.9 | 54.58±39.5 | 55.36±39.9 | 8.18±21.6 | 69.98±35.1 |
|  | Surgery &Chemotherapy | 41.11±35.5 | 6.66±16.1 | 40±39.4 | 16.66±32.3 | 26.66±40.9 | 33.33±35.1 | 26.66±37.8 | 0.0000 | 46.66±47.6 |
|  | Chemotherapy &Radiotherapy | 58.18±31.2 | 21.49±32.8 | 64.91±27.0 | 24.56±30.6 | 53.50±38.3 | 61.40±35.9 | 44.73±43.3 | 17.54±33.5 | 57.01±39.4 |
|  | Surgery, Chemotherapy &Radiotherapy | 41.11±29.3 | 25±27.30 | 40±32.62 | 21.66±27.1 | 31.66±38.2 | 43.33±42.0 | 36.66±43.1 | 8.33±26.2 | 55.±43.62 |
|  | None | 62.23±24.5 | 21.30±30.6 | 64.41±27.4 | 33.58±31.9 | 52.88±37.8 | 56.89±38.4 | 60.90±40.0 | 5.26±19.1 | 73.43±31.7 |
|  | *p-value* | *0.02** | *0.22* | *0.00** | *0.23* | *0.01** | *0.14* | *0.00** | *0.03** | *0.01** |
| Comorbid condition | Yes | 60.45±24.17 | 20.90±30.1 | 62.71 ±29.2 | 27.68± 29.7 | 49.15± 37.3 | 60.45 ±39.8 | 55.36 ±40.4 | 9.60 ±25.5 | 77.96±28.7 |
|  | No | 56.71±28.8 | 21.88± 31.1 | 59.17± 30.8 | 31.40±32.4 | 46.28 ±39.6 | 53.43± 39.1 | 53.71 40.90 | 6.95±21.03 | 67.34±36.2 |
|  | *p- value* | *0.35* | *0.82* | *0.41* | *0.41* | *0.60* | *0.20* | *0.77* | *0.38* | *0.33* |

**Table 3. Mean differences in EORTC QLQ-CX24 functional scale with socio-demographic and clinical characteristics of patients with cervical cancer at TASH, Addis Ababa, Ethiopia.**

| Variables | | Body image | Sexual Activity | Sexual enjoyment | Sexual/vaginal functioning |
| --- | --- | --- | --- | --- | --- |
| Age(years) | 25-54 | 46.03±39.7 | 8.15±21.8 | 41.66±29.3 | 58.59±30.1 |
|  | 55-64 | 56.48±37.5 | 5±19.15 | 55.55±33.3 | 85.18±17.0 |
|  | >65 | 54.52±35.2 | 0.00 | 0.00 | 0.00 |
|  | *p*-value | 0.04* | 0.02* | 0.16 | 0.23 |
| Marital status | Single | 53.33±50.5 | 41.66±5 | 66.6±47.1 | 25±11.0 |
|  | Married | 46.98±39.2 | 10.15±24.4 | 43.8±30.1 | 66.66±29.3 |
|  | Divorced | 52.49±37.4 | 0.4831±4.0 | 33.3±0 | 58.3±0.0 |
|  | Widowed | 54.22±38.32 | 1.13±8.6 | 0.00 | 0.00 |
|  | *p*-value | 0.04* | 0.00* | 0.56 | 0.15 |
| Level of education | Can’t read and write | 48.12±38.3 | 4.16±16.0 | 43.93±29.7 | 57.19±29.1 |
|  | Informal | 52.77±43.25 | 2.38±12.59 | 0.00 | 0.00 |
|  | Primary | 52.38±36.9 | 7.93±24.2 | 26.66±27.8 | 71.66±19.1 |
|  | Secondary | 60.36±38.4 | 11.71±27.4 | 61.90±29.9 | 86.90±17.2 |
|  | Higher | 54.86±43.7 | 29.16±34.15 | 42.85±31.7 | 59.52±39.2 |
|  | *p*-value | 0.43 | 0.00* | 0.26 | 0.12 |
| AMHI in Ethiopian Birr | ≤600 | 50.84±39.5 | 4.14±16.9 | 46.15±37.3 | 64.10±30.1 |
|  | >600 | 49.53±38.0 | 8.02±21.9 | 44.04±27.2 | 64.58±30.1 |
|  | *p*-value | 0.74 | 0.55 | 0.84 | 0.96 |
| Time since diagnosis (months) | <12 | 63.76±24.4 | 5.61±19.01 | 50.72±33.1 | 63.76±24.4 |
|  | 13-60 | 66.11±34.2 | 6.20±19.4 | 35.55±23.4 | 66.11±34.2 |
|  | >60 | 91.66±11.78 | 13.88±33.2 | 66.66 | 91.66±11.7 |
|  | *P*-value | *0.06* | *0.36* | *0.19* | *0.42* |
| Stage of cancer | Unknown | 47.47±40.08 | 0.00 | 0.00 | 0.00 |
|  | Stage 1 | 70±36.6 | 6.66±14.05 | 83.33±11.7 | 33.33±0.00 |
|  | Stage 2 | 46.25±39.2 | 10.59±24.9 | 65.15±32.0 | 36.36±25.00 |
|  | Stage 3 | 54.01±36.2 | 3.39±15.0 | 45.83±25.6 | 72.22±32.7 |
|  | Stage 4 | 49.80±39.7 | 4.19±17.1 | 68.33±27.9 | 46.66±35.8 |
|  | *P*-value | 0.10 | 0.04* | 0.07 | 0.36 |
| Current treatment | Surgery | 60.74±38.4 | 4.44±17.2 | 66.66 | 75±29.4 |
|  | Chemotherapy | 52.77±40.0 | 0.00 | 35.18±24.1 | 0.00 |
|  | Radiotherapy | 49.77±38.5 | 5.45±17.6 | 66.66 | 50.92±29.4 |
|  | Surgery &Chemotherapy | 61.11±46.9 | 16.66±36.0 | 77.77±34.42 | 100±2 |
|  | Chemotherapy &Radiotherapy | 56.72±37.7 | 11.40±28.2 | 55.55±27.2 | 77.77±23.3 |
|  | Surgery, Chemotherapy &Radiotherapy | 47.22±39.7 | 20.00 | 29.62 | 73.61±38.1 |
|  | None | 47.61±38.6 | 3.50±14.36 | 96±26.0 | 71.29±22.4 |
|  | *P*-value | 0.71 | 0.00 | 0.01 | 0.18 |
| Comorbid condition | Yes | 42.74 ±39.3 | 5.08±17.30 | 33.33 ± 33.33 | 69.44± 9.62 |
|  | No | 51.71 ±38.5 | 6.29 ±20.10 | 45.61 ± 30.43 | 64.03 ±30.82 |
|  | *P value* | 0.10 | 0.39 | 0.50 | 0.76 |

**Table 4: Mean differences of EORTC QLQ-CX24 symptom scale with socio-demographic and clinical characteristics of patients with cervical cancer at TASH, Addis Ababa, Ethiopia.**

| **Variables** | | Symptom experience | Lymphedema | Peripheral neuropathy | Menopausal symptoms | Sexual worry |
| --- | --- | --- | --- | --- | --- | --- |
| **Age(in years)** | 25-54 | 43.57±22.0 | 13.04±27.4 | 44.92±38.0 | 57.68±34.9 | 40.72±46.9 |
|  | 55-64 | 42.32±24.2 | 13.05±29.7 | 40±40.90 | 56.38±35.5 | 25.27±40.7 |
|  | >65 | 39.05±20.2 | 10.49±24.9 | 36.41±40.5 | 46.29±37.9 | 20.98±40.0 |
|  | *p-value* | 0.41 | 0.82 | 0.27 | 0.11 | 0.01 |
| **Marital status** | Single | 23.03±18.8 | 13.33±18.2 | 73.33±27.8 | 33.33±47.1 | 80±44.7 |
|  | Married | 44.34±23.5 | 10.31±25.9 | 43.96±39.5 | 57.46±34.5 | 41.74±46.3 |
|  | Divorced | 42.51±20.2 | 17.39±30.0 | 44.44±36.9 | 60.38±32.9 | 27.53±43.8 |
|  | Widowed | 39.80±21.4 | 13.96±29.4 | 36.18±40.2 | 50.14±38.0 | 20.51±39.1 |
|  | *p-value* | 0.77 | 0.29 | 0.89 | 0.89 | 0.00* |
| **Level of education** | Can’t read and write | 44.47±23.0 | 12.45±27.1 | 43.53±39.5 | 58.00±35.5 | 31.07±44.5 |
|  | Informal education | 41.66±20.9 | 3.57±13.8 | 41.66±36.9 | 53.57±37.7 | 32.14±47.5 |
|  | Primary education | 38.16±20.8 | 16.66±33.9 | 44.44±41.4 | 54.76±36.6 | 38.09±47.4 |
|  | Secondary education | 40.37±20.4 | 17.11±33.9 | 32.43±35.5 | 45.04±34.4 | 35.13±41.5 |
|  | Higher education | 28.03±19.87 | 12.5±20.63 | 39.58±42.54 | 47.91±32.13 | 62.5±43.67 |
|  | *p-value* | 0.03* | 0.30 | 0.59 | 0.25 | 0.92 |
| **AMHI in Ethiopian Birr** | ≤600 | 37.6 ± 23.1 | 18.9 ± 21.4 | 29.0 ± 28.1 | 27.2 ± 40.8 | 37.6 ± 23.1 |
|  | >600 | 32.4 ± 22.2 | 18.2 ± 23.3 | 22.9 ± 23.3 | 26.8 ± 40.1 | 32.4 ± 22.2 |
|  | *p-value* | 0.51 | 0.81 | 0.60 | 0.34 | 0.67 |
| **Time since diagnosis (in month)** | <12 | 44.35±22.2 | 11.36±25.3 | 42.01±38.8 | 57.98±35.3 | 35.88±45.59 |
|  | 13-60 | 41.58±21.9 | 15.64±31.9 | 44.61±40.3 | 53.33±35.1 | 28.46±43.5 |
|  | >61 | 15.90±19.7 | 11.11±29.5 | 19.44±30.0 | 30.55±38.8 | 30.55±43.7 |
|  | *p-value* | 0.00* | 0.35 | 0.10 | 0.22 | 0.29 |
| **Stage of cancer** | Unknown | 29.09±40.5 | 13.33±29.8 | 13.33±29.8 | 13.33±29.8 | 40±54.7 |
|  | Stage 1 | 22.72±14.30 | 13.33±32.20 | 60±34.42 | 53.33±42.16 | 40±46.61 |
|  | Stage 2 | 39.22±22.2 | 13.43±28.4 | 42.63±40.1 | 55.29±35.4 | 37.46±45.4 |
|  | Stage 3 | 44.89±22.6 | 12.34±26.0 | 36.11±37.8 | 55.24±35.6 | 29.01±43.9 |
|  | Stage 4 | 46.21±21.1 | 12.96±28.9 | 47.68±39.5 | 58.33±35.1 | 31.48±44.7 |
|  | *p-value* | 0.03* | 0.93 | 0.04 | 0.15 | 0.32 |
| **Current Treatment** | Surgery | 25.05±23.3 | 6.66±18.6 | 33.33±33.3 | 33.33±41.7 | 28.88±43.4 |
|  | Chemotherapy | 50.37±20.0 | 14.58±29.7 | 29.16±36.2 | 56.25±33.8 | 31.25±42.9 |
|  | Radiotherapy | 41.02±22.1 | 14.61±31.1 | 43.85±40.3 | 53.80±34.5 | 34.11±45.0 |
|  | Surgery &Chemotherapy | 25.45±25.1 | 10±22.4 | 36.66±36.6 | 36.66±42.8 | 30±48.3 |
|  | Chemotherapy &Radiotherapy | 41.14±24.8 | 14.91±30.7 | 39.47±39.4 | 63.15±34.4 | 17.54±33.5 |
|  | Surgery, Chemotherapy &Radiotherapy | 27.42±18.3 | 10±24.4 | 56.66±34.3 | 50±38.2 | 58.73±49.3 |
|  | None | 49.69±19.8 | 10.77±23.7 | 41.85±39.7 | 60.65±34.7 | 34.33±46.2 |
|  | *P*-value | *0.37* | *0.05** | *0.05** | *0.45** | *0.06* |
| **Comorbid conditions** | Yes | 40.31 ±20.5 | 18.64± 31.1 | 46.32± 39.5 | 55.36±36.9 | 33.89±44.8 |
|  | No | 43.00± 22.8 | 11.72± 27.0 | 41.56±39.5 | 55.71±35.4 | 33.23±44.9 |
|  | *p-value* | 0.39 | 0.77 | 0.39 | 0.94 | 0.91 |
